# A temporal network analysis of drug co-prescription during antidepressants and anxiolytics dispensing in the Netherlands from 2018 to 2022

**DOI:** 10.1016/j.gloepi.2026.100248

**Published:** 2026-01-14

**Authors:** Aly Lamuri, Spyros Balafas, Eelko Hak, Jens H. Bos, Frederike Jörg, Talitha L. Feenstra

**Affiliations:** a University of Groningen, Groningen, Netherlands; b University of Indonesia, Depok, Indonesia; c University Medical Centre Groningen, Groningen, Netherlands

**Keywords:** Graph theory, Prescription registry, Co-prescription, Mental health, Public health monitoring

## Abstract

**Background:**

Drug prescription networks (DPNs) model the temporal dynamics of medication co-prescription within a population. Understanding these networks can provide insights into polypharmacy and prescribing behaviors.

**Objective:**

This study assesses the structural characteristics of temporal DPNs derived from daily co-prescriptions of antidepressants, anxiolytics, and other therapeutic drug classes. By analyzing these networks using eigenvector centrality, we identify influential medications and prescribing patterns.

**Methods:**

We utilized the IADB.nl database, including prescriptions from 128 Dutch pharmacies (2018–2022). A cohort of patients prescribed antidepressants/anxiolytics was extracted. Medications were classified using the Anatomical Therapeutic Chemical (ATC) system into 24 therapeutic classes. Time-varying DPNs were constructed as undirected graphs using symmetric daily dose-adjusted co-prescriptions. Eigenvector centrality ($c_{e}$) quantified relative nodal importance. Weekly-aggregated data included number of dispensing ($n_{c}$) and eigenvector centrality, which were decomposed using a singular-spectrum approach.

**Results:**

Antidepressants ($c_{e}$: 0.09, $n_{c}$: 28,993) and anxiolytics ($c_{e}$: 0.05, $n_{c}$: 14,061) had high eigenvector centrality, demonstrating frequent co-prescription. Other ATC groups with high centrality included those for the alimentary tract and metabolism (A01-A16), blood and blood-forming organs (B01-B06), cardiovascular system (C01-C10), respiratory system (R01-R07), and analgesics (N02).

**Discussion:**

DPNs revealed key co-prescription patterns. High-centrality medications highlight potential targets for drug monitoring, such as identifying co-prescription trends that may warrant evaluation for safety, appropriateness, or policy oversight. This approach aids in identifying influential medications and refining prescribing oversight.

## Highlights

• Multi-class co-prescription was over tenfold more common than same-class use
• Seven central drug classes bridge psychiatric and chronic disease treatments
• Network analysis identifies prescribing hubs missed by standard utilization methods
• Results align with Dutch guidelines on anxiety and depression treatment
• Co-prescription patterns mirror multimorbidity in psychiatric and chronic care

## Introduction

The complexity of health-related phenomena has led to the growing application of network analysis in medicine and epidemiology [1], [2]. Graph theory provides the mathematical foundation for network analysis, offering an approach to study the relationships among the entities of a complex system. The early implementations of network analysis focused primarily on transmission, social, and organizational networks. More recently, Cavallo et al. [3] introduced drug prescription networks (DPNs) to model population-level drug prescription data. Bazzoni et al. [4] formalized the term, emphasizing its utility in understanding polypharmacy.

Psychiatric polypharmacy is defined as a patient with at least two concurrent psychiatric medications [5]. With a prevalence between 13%–90%, psychiatric polypharmacy manifests in five categories, namely same-class, multi-class, adjunctive, augmentation, and total polypharmacy [5]. Same-class polypharmacy is the use of multiple medications from the same class. Multi-class polypharmacy is the use of multiple medications from different classes indicated for the same symptom cluster. Adjunctive polypharmacy is the use of additional medications to treat side effects due to other medications. Augmentation polypharmacy is the use of full-dose and sub-dose medications from a different class for the same symptom cluster. Finally, total polypharmacy is the overall number of medications a patient uses, including those prescribed for multiple psychiatric symptom clusters as well as for comorbid non-psychiatric conditions [5]. For policymakers, differences in defining polypharmacy pose a challenge to design an effective monitoring system [6]. Such monitoring is essential to identify prevalent co-prescription pattern that may impact treatment appropriateness and healthcare policy development.

A DPN is a graphical representation where medications correspond to nodes, and edges represent co-prescription relationships between them. Typically, simple undirected graphs are represented by a square symmetric adjacency matrix A, where each nonzero element $A_{ij}$ indicates that medications i and j were co-prescribed on a given day. A population-level DPN can be constructed by aggregating individual patient networks through matrix addition [3]. Analyzing the structural characteristics of a DPN using both local (medication-level) and global (network-level) graph measures provides insights into co-prescription dynamics.

A DPN for an individual on a given day can be formalized as a simple undirected graph where medications correspond to nodes, and edges connecting pairs of nodes represent co-prescription relationships between medications [3]. Typically, simple undirected graphs are represented by a square symmetric matrix with zeros on the diagonal called the adjacency matrix [7]. In the adjacency matrix of the individual-level DPN, nonzero off-diagonal elements correspond to two medications being co-prescribed on a given day.

A population-level DPN on a given day can be constructed from individual-level DPNs through matrix addition of the corresponding adjacency matrices [3]. A DPN at the population level on a given day is then formulated as a weighted undirected graph, which is characterized by a weighted adjacency matrix with weights equal to the counts of medication co-prescription occurrences. Therefore, analyzing the structural characteristics of a DPN over time using both local (medication-level) and global (network-level) graph measures offers valuable insights into co-prescription dynamics and can deepen our understanding of polypharmacy.

Local network statistics, such as node centrality measures, quantify the importance of a medication in polypharmacy patterns [8]. Addressing concerns about standardized polypharmacy indicators raised by Sirois et al. [6] and Delara et al. [9], we highlight eigenvector centrality as a key measure of a medication’s connection to highly prescribed drugs. In this context, eigenvector centrality offers a useful descriptive measure because it captures not only the number of co-prescriptions involving a medication, but also the connectedness of the medications with which it is co-prescribed. Medications with high eigenvector centrality are therefore structurally embedded within densely connected prescribing patterns, reflecting their position within broader co-prescription structures. Building on prior studies [3], [4], this study focuses on eigenvector centrality as a network-based indicator of structural connectedness in co-prescription patterns, rather than as a direct measure of clinical polypharmacy at the individual level. Specifically, we aim to assess the structural characteristics of temporal DPNs derived from dispensing data, with particular emphasis on identifying medication classes that occupy central positions within co-prescription networks among adult patients prescribed antidepressants or anxiolytics.

## Methods

### Source of data and study population

All data in this study originated from the University of Groningen IADB.nl, a dynamic pharmacy database containing prescription records since 1994. The database covers approximately 128 community pharmacies serving over one million patients and was accessed on the 20th of September 2023. In the Netherlands, the primary route of obtaining prescription medications is through community pharmacies, as physicians issue prescriptions that are almost exclusively dispensed in these settings. Consequently, the database provides a comprehensive longitudinal record for each individual, supported by high patient-pharmacy commitment. All patients are recorded in the database, irrespective of health care insurance, where the prescription rates, age, and gender are generalizable to represent the Netherlands [10]. Each record includes the dispensing date, quantity, dose regimen, number of days prescribed, prescribing physician, and the corresponding Anatomical Therapeutic Chemical (ATC) code. Medication dispensed during hospitalization and over-the-counter drugs are not captured, but prescription medication records in community pharmacies are otherwise complete.

This analysis includes daily drug dispensing record from a static cohort of adults aged 18 to 65 years and prescribed anxiolytics or antidepressants at least once in the period 2018–2022. For this study, only a subset of the database was used to construct the DPN: anonymized patient identifiers, age, prescription start and end dates, and ATC codes. No additional individual-level data were included to align with general data minimization requirements. Other demographic or clinical information available in the database was not extracted.

### Graph theory

Mathematically, a simple undirected graph G is a pair $G=\left(V,E\right)$, where $V=\left(1,\ldots ,n\right)$ is the set of nodes and $E=\left(\left\{i,j\right\}\;|\;i,j\in V,\;i\neq j\right)$ is the finite set of edges. The adjacency matrix $A\in {\left\{0,1\right\}}^{n\times n}$ of a simple undirected graph G is a square symmetric matrix of dimension n×n defined as:

(1) $$A_{ij}=\left\{\begin{array}{cc} 1\; & \text{if}\left\{i,j\right\}\in E\\ 0\; & \text{otherwise} \end{array}\right)$$

with elements $A_{ij}=A_{ji}$, for all i,j∈V where i≠j [7].

In our setting, drug co-prescriptions from a single individual p on a specific day t are encoded in a simple undirected graph $G^{p}\left(t\right)=\left(V,E^{p}\left(t\right)\right)$ with n×n adjacency matrix $A^{p}\left(t\right)$. In these daily observed individual-level networks, the node set V corresponds to a set of n medication classes according to the ATC classification, and the edge set $E^{p}\left(t\right)$ contains edges that indicate the concurrent use of pairs of medications for a particular individual p on a given day t.

### Data pre-processing to build the data matrix

Assume an observational study where P individuals indexed by p, p=1,…,P are followed for each day t, t=1,…,T for a total period of T days. To derive the population-level DPN on each day t with adjacency matrix $A_{t}$, the individual-level daily prescription matrices $A^{p}\left(t\right)$ were aggregated by computing their element-wise sum, that is,

(2) $$\overset{P}{\underset{p=1}{\sum }}A^{p}\left(t\right),\;\text{where}\;A\left(t\right)\in \;Z_{+}^{n\times n}.$$

The matrix $A\left(t\right)$ captures the population frequency of co-prescriptions (off-diagonal) and single prescriptions (diagonal) in the data at the specified time t. Each element $A_{ij}\left(t\right)$ in the matrix was interpreted as the number of patients being prescribed medication classes i and j, with i,j∈V, on the same day t.

The population-level DPN is then a weighted undirected graph $G\left(t\right)=\left(V,E\left(t\right)\right)$ observed over T discrete time points t=1,…,T, where: V is the fixed set of n nodes; $E_{t}\subseteq \left\{\left\{i,j\right\}:i,j\in V,i\neq j\right\}$ is the edge set at time t.

The population-level DPN at time t is fully described by its symmetric weighted adjacency matrix $A\left(t\right)\in Z_{+}^{n\times n}$, which is defined as:

$$A_{ij}\left(t\right)=\left\{\begin{array}{cc} w_{t,ij}>0\; & \text{if}\left\{i,j\right\}\in E\left(t\right),\\ 0\; & \text{otherwise}, \end{array}\right)\;\text{with}A\left(t\right)={A\left(t\right)}^{⊤}.$$

In the original work of DPN, all defined daily dose (DDD) were assumed equal to 1, allowing for applying raw counts as the edge weights. However, since not all medications have DDD = 1, this assumption could lead to inaccurate representations. To enhance the accuracy of edge weights, we introduced a weighting function to adjust the contribution of each co-prescription. Specifically, each co-prescribed medication class was assigned a weight $\omega_{j}$ based on its DDD value using a Gaussian kernel centered at the baseline weight $\omega_{B}=1$, i.e., the expected DDD, with standard deviation $\sigma =\frac{1}{3}$ to allow for a gradual reduction in weight as DDD deviates from 1.

(3) $$\omega_{j}=\frac{1}{\sigma \sqrt{2\pi }}e^{-{\frac{\left(DDD_{j}-1\right)}{2\sigma^{2}}}^{2}}$$

The edge weight between medication classes i and j was computed as the average of their individual weights:

(4) $$\overset{\text{Unregularized}}{\left[\begin{array}{ccc} n_{1,1} & \ldots & n_{1,N}\\ \vdots & \ddots & \vdots \\ n_{N,1} & \ldots & n_{N,N} \end{array}\right]}\;\to \;\overset{\text{Regularized by}DDD}{\left[\begin{array}{ccc} \frac{1}{2}\cdot \left(\omega_{1}+\omega_{1}\right) & \ldots & \frac{1}{2}\cdot \left(\omega_{1}+\omega_{N}\right)\\ \vdots & \ddots & \vdots \\ \frac{1}{2}\cdot \left(\omega_{N}+\omega_{1}\right) & \ldots & \frac{1}{2}\cdot \left(\omega_{N}+\omega_{N}\right) \end{array}\right]}$$

This weighting approach scaled the edge weight that deviate substantially from the standard $\omega_{B}=1$, improving comparability across medication classes. Although diagonal elements capture single-class dispensing frequencies, they were excluded from network analyses to avoid self-loops. Because DDD represents a standardized population-level metric rather than individual dosage, our weighting scheme was applied as a descriptive adjustment.

### Centrality measures in graph theory

Centrality measures formalize the identification of important nodes in a graph [7]. Different node centrality measures quantify different structural properties of a node. Previous work on DPNs highlighted four centrality measures that could be used to assess the position of a medication within a co-prescription network [2]. Degree centrality in a DPN describes the number of co-prescription with the medication of interest. High (low) degree centrality means the medication is often (seldom) co-prescribed. Betweenness centrality indicates the frequency of a medication connecting two other medications by the shortest possible path. High (low) betweenness centrality means the medication is (not) a “bridge” between different kind of medications. Closeness centrality is the average distance between one medication to all other medications in the DPN. High (low) closeness centrality means that the medication is (not) commonly co-prescribed. Eigenvector centrality reflects the number of co-prescription with medications that have a prominent network position in the DPN. High (low) eigenvector centrality means that the medication is often (seldom) co-prescribed with other central medications.

The choice of centrality largely depends on the objective of network analysis. As a general guide, degree centrality is useful to identify popular medication and monitor drug overuse. Betweenness centrality is suitable for targeting for drug-interaction study and optimizing therapy plan. Closeness centrality indicates widely-used key medications and efficiency in treatment networks. Eigenvector centrality is helpful to identify influential medications and narrow down high-impact medications for drug monitoring. We defined high and low eigenvector centrality relative to the expected value $C_{e}=\frac{1}{n}$, based on the heuristic reference of uniform connectivity and unweighted network (see “Determining relative importance”). This study focused on eigenvector centrality to evaluate which medications are central on the prescription of antidepressants and anxiolytics. Eq. (5) outlines the calculation of eigenvector centrality; $c_{i}$ and $c_{j}$ are the centralities of nodes $v_{i}$ and $v_{j}$, respectively, with i,j∈V; λ is the eigenvalue; $A_{ji}\left(t\right)$ is an element on row j and column i from an adjacency matrix $A\left(t\right)$, representing the connection between node $v_{j}$ and $v_{i}$ with i,j∈V.

(5) $$c_{i}\left(t\right)=\frac{1}{\lambda }\underset{j\neq i}{\sum }A_{ji}\left(t\right)\cdot c_{j}\left(t\right)$$

### Data analysis

#### Exploratory descriptive analysis

The data was extracted as a daily time series and aggregated into weekly intervals for analysis. It includes four metrics: daily prescription dispensing, daily patients, dispensing-to-patient ratio, and eigenvector centrality. For each metric, a univariate descriptive analysis was performed, reporting the mean, median, standard deviation, and interquartile range to summarize centrality and dispersion.

#### Exploring seasonality in the dataset

Exploration on seasonality was done on detrended daily and weekly data by generating seasonal plots and calculating the autocorrelation (ACF) and partial autocorrelation function (PACF). Seasonal plots were generated for weekly and yearly pattern by using daily and weekly data, respectively. To generate the seasonal plots, the data was first deconstructed based on its period. For daily data, the weekly period was used; while for weekly data, the yearly period was used. The weekly period was obtained by creating an ordered value formatted as year - week, e.g. 2018 - W01, whereas the yearly period was an order from 2018 to 2022. We then grouped the series by its deconstructed period and visually examine seasonality as overlapping pattern in most periods. To substantiate the findings, ACF and PACF plots were used to check on statistical significance of a given pattern.

#### Summarizing co-prescription

This study explored same-class, multi-class, and total co-prescription categories. These three groups were selected as they can be directly inferred from medication dispensing data aggregated at the population level over the year. The statistics were summarized as prescription-day, defined as the number of medications dispensed on a given day. For example, seven medications prescribed for one day account for 7 prescription-days. The mean and standard deviation represented the average number of prescription-days per person for any co-prescription regimen.

Although five polypharmacy categories were described by Kukreja et al. [5], augmentation and adjunctive polypharmacy require clinical context such as diagnoses to determine the rationale for combined prescriptions, which is not available in our data source. Therefore, this study only focused on the three categories that rely solely on medication class information.

#### Decomposition with singular spectrum analysis

Classical and seasonal-trend decomposition techniques may not fully capture complex periodic patterns in time-series data. Singular spectrum analysis (SSA) is a non-parametric method that leverages Hankel matrix embedding for decomposition. In this study, SSA was applied to descriptively identify trends and seasonal components in weekly aggregated prescription data. First, a basic SSA model with the lag parameter L = 52 was used to extract the dominant trend. The residuals were then analyzed with a second SSA model using $L=\frac{N}{2}$ to capture complex periodic patterns. The extracted trend, residuals, and oscillatory components were evaluated visually and statistically, with Mann–Kendall trend tests applied to assess long-term changes. A complete documentation of this approach and its rationale was included in the supplementary file section S1.2.

#### Determining relative importance

Since the sum of all normalized eigenvector centralities $c_{i}$ in Eq. (5) equals 1, the expected eigenvector centrality $C_{e}$ for each node in a uniformly connected network is $\frac{1}{n}$, where n is 24, representing the total number of nodes. In such a network, each node has an equal probability of being connected to any other, and thus no node is more “central” than another. This expected value, $\frac{1}{24}$, serves as a baseline reference of uniform connectivity and unweighted network. Nodes with $c_{i}$ greater than $\frac{1}{24}$ were categorized as having high eigenvector centrality, and those below the expected value were considered low.

#### Subgroup analysis

Subgroup analyses were conducted by first categorizing the population into two age groups: <65 and ≥65 years [11]. The second categorization was based on general polypharmacy, defined as receiving ≥5 medications for at least 30 consecutive days [12]. These subgroup classifications were combined to form three comparison pairs: <65 vs. ≥65 years; non-polypharmacy vs. polypharmacy; and the non-polypharmacy group aged <65 years vs. the polypharmacy group aged ≥65 years. Separate population-level DPNs were constructed independently for each subgroup using the same pre-processing and weighting procedures to calculate its respective eigenvector centrality.

## Results

### Description of the population

IADB recorded 149,071 patients with at least one dispensing of antidepressants or anxiolytics within five years of data extraction. Table 1 captures the demographical dynamics of the population from 2018 to 2022. The ratio of male to female only varied slightly, and the average age steadily increased over the year. These findings imply that the population demography stays relatively stable overtime without any indication of sudden changes. Importantly, the COVID-19 pandemic in 2020 did not substantially influence dispensing volumes because the community pharmacy remained in service as a part of nationwide policy. There was no clear change in prescription trends during and after the pandemic, with an average of 2,230,028 in annual dispensing for all medication classes (standard deviation/SD: 47,478, interquartile range/IQR: 64,009).

**Table 1:** Number of participating patients with at least one dispensing of antidepressants or anxiolytics from 2018 to 2022.

Year	Number of patients (%)			Mean age (SD)^a^	
	Male	Female	Total	Male	Female
2018	42,164 (36.6%)	73,124 (63.4%)	115,288	48.72 (12.33)	48.02 (12.80)
2019	42,427 (36.7%)	73,109 (63.3%)	115,536	49.26 (12.62)	48.54 (13.02)
2020	42,010 (36.6%)	72,700 (63.4%)	114,710	49.77 (12.79)	49.15 (13.16)
2021	42,016 (36.7%)	72,532 (63.3%)	114,548	50.15 (13.04)	49.55 (13.42)
2022	39,464 (36.7%)	67,930 (63.3%)	107,394	50.82 (13.12)	50.38 (13.50)

a SD: Standard Deviation

### Cyclicality and seasonality of medication dispensing

Visual inspection of daily dispensing volumes revealed consistent weekly cycles. Dispensing peaked on Monday, with an average of 7222 [SD: 999.79] and dropped to its lowest on Saturday, with an average of 2808 [SD: 174.08] dispensing. The variation between weekends and weekdays showed a regular and repeating structure in the data, as shown in Table 2. The cyclical patterns are detailed in the supplementary material section S2.1. Seasonality was explored as dependencies at daily and weekly lags and further described in the supplementary material section S2.2 and S2.3.

**Table 2:** Daily dispensing from 2018 to 2022 among patients with at least one dispensing of antidepressants or anxiolytics.

Day	Number of prescriptions dispensed		
	Mean (SD)^a^	Median (IQR)^b^	Range
Monday	7222 (999.79)	7400 (524.00)	2935–9194
Tuesday	6793 (640.76)	6821 (483.00)	3164–8620
Wednesday	6865 (601.11)	6852 (566.00)	3915–8503
Thursday	7109 (592.11)	7136 (579.00)	4156–8608
Friday	6726 (535.52)	6771 (597.00)	3589–8712
Saturday	2808 (174.08)	2803 (169.00)	2293–4533
Sunday	2991 (166.44)	3010 (161.25)	2381–3478

a SD: Standard Deviation.

b IQR: Interquartile Range.

### Co-prescription in the population

Table 3 outlines the type of co-prescription in the population and reports two statistics: the sum and the mean with its standard deviation. The sum indicates the total prescription-days of co-prescription within a year, and the mean with its standard deviation represents the average number of co-prescription days per person. For example, in 2018, same-class co-prescription of antidepressants occurred for a total of 73,528 prescription-days across the population, with an average of 0.06 prescription-days per person, interpreted as:

“For every 100 people treated with antidepressants, there were approximately 6 days in total during which same-class co-prescription regimens occurred”.

While these average numbers per person may appear low, they are not intended to reflect the absolute epidemiological importance of co-prescriptions. Instead, prescription-days serve as a relative metric to compare the extent of co-prescription across categories and over time. For instance, Table 3 shows that multi-class polypharmacy generally exhibits higher prescription-day values than same-class polypharmacy among individuals prescribed antidepressants. Therefore, prescription-day counts should be interpreted in the context of relative prescribing patterns, rather than as a direct indicator of epidemiological significance.

**Table 3:** Summary statistics of different psychiatric co-prescription categories.

Year	Medication	Population level^a^		Per person (Mean [SD])^b^		Total co-prescription^c^	
		Same-class	Multi-class	Same-class	Multi-class	N	Mean [SD]
2018	Antidepressants	73,528	844,789	0.06 [0.25]	0.69 [1.17]	7,264,585	5.90 [4.18]
2019	Antidepressants	75,794	821,726	0.06 [0.25]	0.65 [1.13]	7,489,533	5.92 [4.17]
2020	Antidepressants	74,924	828,043	0.06 [0.25]	0.65 [1.13]	7,564,952	5.93 [4.15]
2021	Antidepressants	75,549	804,264	0.06 [0.25]	0.64 [1.12]	7,466,014	5.91 [4.17]
2022	Antidepressants	74,026	797,843	0.06 [0.25]	0.64 [1.12]	7,474,032	5.95 [4.17]
2018	Anxiolytics	34,122	1,049,590	0.03 [0.17]	0.85 [1.21]	7,264,585	5.90 [4.18]
2019	Anxiolytics	31,955	1,044,683	0.03 [0.16]	0.83 [1.18]	7,489,533	5.92 [4.17]
2020	Anxiolytics	32,249	1,053,849	0.03 [0.16]	0.83 [1.17]	7,564,952	5.93 [4.15]
2021	Anxiolytics	29,912	1,035,388	0.02 [0.16]	0.82 [1.16]	7,466,014	5.91 [4.17]
2022	Anxiolytics	31,527	1,027,019	0.03 [0.16]	0.82 [1.16]	7,474,032	5.95 [4.17]

Prescription day is defined as the number of dispensed medication per day.

Multi-class co-prescription is defined as the co-prescription of medications from different ATC classification.

a The total occurrence of co-prescription on the population level/prescription-day.

b SD: Standard Deviation; The expected occurrence of co-prescription per person/prescription-day.

c The overall number of medications a patient uses, including those prescribed for multiple psychiatric symptom clusters as well as for comorbid non-psychiatric conditions.

### Time-series decomposition

**Fig. 1:**
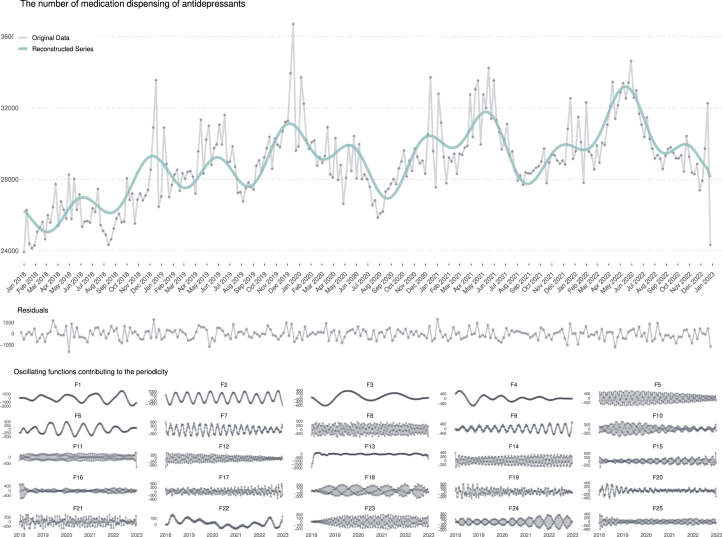
Singular spectrum analysis (SSA)-based decomposition of antidepressant dispensing volumes 2018–2022. The SSA separated the original time-series into the overall trend, residuals, and 25 oscillatory components (F1-F25). The green line represents the reconstructed time-series using the trend component, F1, and F2, highlighting the primary pattern in the series.

For brevity, Fig. 1 only depicts the decomposition result of antidepressants dispensed over the years. SSA-based decomposition separates the trend from its harmonics, as shown in the lower half of the plot. The trend explains roughly 90% of the variability, and harmonics explain 10%. The first two harmonics, F1 and F2, captured most of the variability in a series compared to the rest of it. The trend, F1, and F2 were used to reconstruct the time series. The largest panel in Fig. 1 displays the reconstructed time-series (green line) overlaid on the original data points (gray line).

Additional decomposition results for other medication classes are presented in the supplementary section S2.4. Broadly, antidepressants exhibited a steady increasing trend over time, while anxiolytics showed a gradual decline both in dispensing frequency and eigenvector centrality. Notably, the decomposition made long-term directional patterns more discernible across all medication classes, emphasizing the utility of SSA in isolating interpretable temporal dynamics from complex dispensing data. These decomposed trends provided the reconstructed data for the subsequent analysis.

### Eigenvector centrality measures

**Fig. 2:**
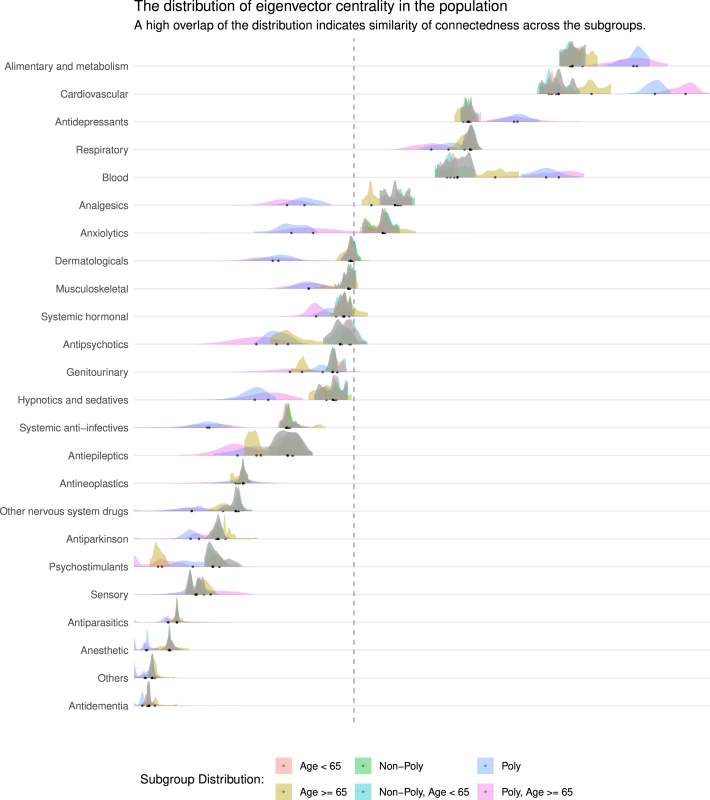
Clusters of medications showing significant co-prescription patterns for antidepressnts and anxiolytics. The vertical dashed line represents a reference value corresponding to equal connectedness across medication classes. This is used for identifying medication classes with eigenvector centrality exceeding the threshold. The gray density area represents eigenvector centrality in the population, while the colored density areas represent the subgroups.

Seven medication classes exhibited high centrality, as shown in Fig. 2 and Table 4. Notably, highly dispensed medications generally had high eigenvector centrality, consistent with the definition of eigenvector centrality in Eq. (5). However, this relationship was not strictly proportional. For example, although antidepressants had a higher number of prescription dispensed compared to medications for the respiratory system (6,108,776 vs 5,492,900), they exhibited lower eigenvector centrality (9.48e−2 [SD: 2.24e−3] vs 9.53e−2 [SD: 2.21e−3]). A similar pattern was observed for anxiolytics, which had more dispensing than analgesics (2,644,309 vs 2,557,528) but lower eigenvector centrality (5.25e−2 [SD: 4.13e−3] vs 5.84e−2 [SD: 3.94e−3]). These discrepancies show that higher centrality scores capture more than just a high dispensing volume.

The distribution of eigenvector centralities exhibited a central peak at intermediate values and heavy tails at both extremes, reflecting the temporal fluctuations of node influence. This pattern arose from the recursive nature of eigenvector centrality, where each node’s importance depended on the importance of its neighbors. Aggregating centrality value over time captured the evolving dynamics of the network, where nodes occasionally attained a relatively higher or lower centrality. In contrast, the width of the distribution remained relatively stable, indicating that many co-prescription connections persisted consistently over time.

The eigenvector centrality patterns shown in Fig. 2 also highlight the distribution of several subgroups, each distinguished by color. Most subgroup distribution largely overlapped with the general population, represented by the gray density curves. However, two subgroups consistently diverged from this overall pattern: the polypharmacy group and the subset of polypharmacy patients aged ≥65 years. These subgroups exhibited higher eigenvector centralities across four medication classes, indicating denser co-prescription structures within these groups. In addition, the ≥65 years subgroup showed elevated eigenvector centralities in three medication classes, although its magnitude was smaller than that observed in the broader polypharmacy population.

**Table 4:** Descriptive statistics of dispensing data from 2018 to 2022, ordered by Eigenvector centrality. Centrality values represent averages over the study period.

			Mean [SD]^a^	
	Dispensing	Centrality	DDD^b^	Weight^c^
Alimentary and metabolism	11,512,424	1.59e−1 [5.00e−3]	5.97e−1 [3.26e−1]	7.28e−1 [2.06e−1]
Cardiovascular	9,778,030	1.50e−1 [6.87e−3]	5.38e−1 [3.05e−1]	6.92e−1 [2.00e−1]
Respiratory	5,492,900	9.53e−2 [2.21e−3]	6.55e−1 [3.25e−1]	7.70e−1 [2.15e−1]
Antidepressants	6,108,776	9.48e−2 [2.24e−3]	5.36e−1 [3.09e−1]	6.92e−1 [2.03e−1]
Blood	2,908,944	8.87e−2 [5.64e−3]	6.93e−1 [3.91e−1]	7.78e−1 [2.13e−1]
Analgesics	2,557,528	5.84e−2 [3.94e−3]	4.23e−1 [2.60e−1]	6.16e−1 [1.66e−1]
Anxiolytics	2,644,309	5.25e−2 [4.13e−3]	4.63e−1 [2.88e−1]	6.35e−1 [1.73e−1]
Dermatologicals	2,086,095	4.03e−2 [1.34e−3]	8.08e−1 [3.39e−1]	8.62e−1 [2.09e−1]
Musculoskeletal	1,449,535	3.95e−2 [1.52e−3]	5.59e−1 [3.26e−1]	7.10e−1 [2.11e−1]
Systemic hormonal	1,602,716	3.80e−2 [1.71e−3]	4.97e−1 [2.76e−1]	6.55e−1 [1.66e−1]
Antipsychotics	2,473,609	3.70e−2 [3.20e−3]	4.04e−1 [2.65e−1]	6.10e−1 [1.69e−1]
Genitourinary	2,099,833	3.39e−2 [9.53e−4]	7.46e−1 [3.69e−1]	8.29e−1 [2.12e−1]
Hypnotics and sedatives	1,467,752	3.37e−2 [3.23e−3]	6.31e−1 [3.16e−1]	7.49e−1 [2.09e−1]
Systemic anti-infectives	734,706	2.07e−2 [1.47e−3]	6.34e−1 [3.23e−1]	7.64e−1 [2.21e−1]
Antiepileptics	1,078,266	2.06e−2 [2.73e−3]	4.29e−1 [2.52e−1]	6.25e−1 [1.66e−1]
Antineoplastics	449,030	1.03e−2 [4.68e−4]	6.13e−1 [3.01e−1]	7.49e−1 [2.05e−1]
Other nervous system drugs	362,624	8.79e−3 [1.08e−3]	5.23e−1 [3.16e−1]	6.78e−1 [1.93e−1]
Antiparkinson	306,541	6.17e−3 [5.64e−4]	3.53e−1 [2.21e−1]	5.74e−1 [1.37e−1]
Psychostimulants	459,207	5.56e−3 [8.26e−4]	5.20e−1 [3.13e−1]	6.74e−1 [1.90e−1]
Sensory	196,513	3.36e−3 [5.91e−4]	4.66e−1 [2.87e−1]	6.37e−1 [1.80e−1]
Antiparasitics	88,760	1.61e−3 [1.20e−4]	5.24e−1 [2.53e−1]	6.72e−1 [1.65e−1]
Anesthetic	39,455	1.15e−3 [2.15e−4]	6.45e−1 [4.47e−1]	7.39e−1 [2.31e−1]
Others	18,157	2.94e−4 [4.86e−5]	4.55e−1 [2.73e−1]	6.37e−1 [1.77e−1]
Antidementia	9937	2.00e−4 [4.76e−5]	6.80e−1 [2.85e−1]	7.87e−1 [2.02e−1]

a SD: Standard Deviation.

b Defined Daily Dose, representing the assumed average maintenance dose per day for a drug used for its main indication in adults (WHO definition).

c Edge weight assigned to each medication class in the drug prescription network, reflecting the strength of co-prescription connections (see Methods: Data pre-processing to build the data matrix).

## Discussion

This network analysis of dispensing data in individuals using antidepressants or anxiolytics revealed distinct population-level patterns. While same-class co-prescription was more common among antidepressant users, multi-class co-prescription predominated among those receiving anxiolytics. Notably, in both groups, multi-class co-prescription was at least ten times more prevalent than same-class co-prescription. Seven medication classes consistently exhibited high eigenvector centrality, indicating that these classes occupied structurally central positions within the co-prescription network. These include medications for the alimentary tract and metabolism (ATC code A), blood and blood-forming organs (B), cardiovascular system (C), respiratory system (R), and analgesics (N02). Importantly, high eigenvector centrality reflects structural connectedness within the prescribing network, rather than clinical influence or treatment intent. Medication classes with high centrality were frequently co-prescribed with other highly connected classes, linking psychiatric and non-psychiatric medications within broader dispensing patterns. Such central positioning likely reflects the co-occurrence of psychiatric treatment with the pharmacological management of chronic conditions and comorbidities, rather than specific prescribing strategies directed at psychiatric symptoms alone.

The predomination of multi-class co-prescription among anxiolytics recipients is consistent with the recommendations in the Dutch College of General Practitioner (*Nederlands Huisartsen Genootschap*/NHG) [13]. Specifically, the NHG guideline for anxiety disorders advises short-term benzodiazepine use, limited to two to four weeks, as an adjunct to Selective Serotonin Reuptake Inhibitors (SSRIs) during treatment initiation. In contrast, the NHG guideline for depressive disorders recommends initiating pharmacotherapy with a single SSRI and discourages the combination of multiple antidepressants [14]. These findings are consistent with evidence-based clinical recommendations and are unlikely to be solely attributable to sampling or measurement artifacts.

Our findings also align with previous research showing that antidepressants and anxiolytics are frequently co-prescribed with other medications [5], and that multi-class regimens contribute most to psychopharmaca polypharmacy [15]. Additionally, our study offers a more granular view of these patterns by characterizing their network properties. By distinguishing between same-class and multi-class co-prescriptions and evaluating medication centrality, our analysis highlights that certain medication classes consistently occupy central positions within the prescribing network. These characteristics are consistent with the high prevalence of multimorbidity in populations treated for depression and anxiety, where associations between psychiatric conditions and chronic illnesses are well documented [16]. Conditions such as diabetes mellitus, thyroid disorders, and asthma have been linked to an increased risk of depression [17]. The need for long-term pharmacological management further drives co-prescription patterns. Additionally, chronic illness-related anxiety can contribute to heightened prescribing of anxiolytics [18]. This underscores the interconnected nature of psychopharmaca prescribing patterns and highlights the complexity of medication management in these populations.

DPN complements traditional drug utilization analyses by capturing the structural properties of prescribing behaviors. Beyond evaluating individual drug utilization or basic pairwise co-prescriptions, DPN provides a structural perspective by mapping the interconnections between medications within a broader prescribing network [4]. The network approach is a particularly useful method for understanding complex prescribing dynamics, which allows for the identification of central medication classes that influence co-prescription patterns. The high centrality of certain medication classes suggests they serve as critical connectors in treatment regimens, providing the basis for further monitoring and evaluation.

By mapping the relationships between medications, DPN enables the detection of patterns that would be challenging to observe through standard statistical methods. For instance, the high centrality of certain medication classes suggests that they occupy central positions within multi-class co-prescription structures, potentially reflecting their frequent co-prescription in the management of both psychiatric and non-psychiatric conditions. As such, network analysis is a suitable approach for generating hypotheses for further causal or predictive studies, which has also been thoroughly discussed by Askar et al. [8].

We acknowledge several limitations in this study. First, data aggregation at the population level resulted in the loss of individual-level information, limiting interpretation to population trends. Second, while the adjustment of weights based on DDD introduces a novel element to the analysis, it is important to note that DDD values are not always equal to one, as defined by the WHO ATC system. Our study adopted a DDD-based weighting approach to refine network edges, aligning with the recommendation of Cavallo et al. [3]. The adjusted weighting penalizes values deviating significantly from 1, reducing their impact on the network. Future research should explore alternative weighting techniques, such as patient-level dosage adjustments, to enhance network precision.

Despite the limitations, our study demonstrates the utility of DPN as a powerful, data-driven approach to analyzing medication dispensing data. By modeling intricate co-prescription patterns, DPN filters medication classes based on network properties, offering insights into prescribing behaviors. These insights can reveal important trends in medication use, which can be useful to pharmaceutical reviews and public health monitoring. By capturing the complexity of drug prescription relationships, DPN holds significant potential to improve decision-making in both clinical and administrative contexts.

Future DPN studies on individuals using antidepressants or anxiolytics should adopt a more fine-grained ATC classification to identify specific medications driving co-prescription patterns within the currently identified seven medication classes. A more granular approach could reveal whether certain drugs disproportionately contribute to multi-class co-prescription and whether these prescribing trends vary across patient demographics or healthcare settings. Such insights could refine our understanding of prescribing behaviors and inform targeted interventions for optimizing medication regimens.

## Conclusion

This study demonstrates the usefulness of drug prescription networks (DPNs) for characterizing population-level co-prescription patterns among individuals prescribed antidepressants or anxiolytics. We observed that multi-class co-prescription regimens were substantially more prevalent than same-class co-prescription, particularly among users of anxiolytics, consistent with existing clinical practice guidelines.

By applying network-based measures, we identified seven medication classes that consistently occupied structurally central positions within the co-prescription network. These central positions reflect frequent co-prescription with other highly connected medication classes and are indicative of broader prescribing structures that integrate psychiatric treatments with medications commonly used for chronic somatic conditions. Such patterns are consistent with the high burden of multimorbidity in populations treated for depression and anxiety.

## CRediT authorship contribution statement

**Aly Lamuri:** Writing – original draft, Visualization, Project administration, Methodology, Formal analysis, Conceptualization. **Spyros Balafas:** Writing – review & editing, Methodology. **Eelko Hak:** Writing – review & editing. **Jens H. Bos:** Project administration, Investigation, Data curation. **Frederike Jörg:** Writing – review & editing, Writing – original draft, Supervision, Conceptualization. **Talitha L. Feenstra:** Writing – review & editing, Writing – original draft, Supervision, Methodology, Conceptualization.

### Funding

This work was supported by The Indonesia Endowment Funds for Education (LPDP) in the form of a PhD scholarship to AL with Grant Agreement Number 0007457/PHA/D/2/lpdp2022 (26 July 2025).

## Declaration of competing interest

The authors declare that they have no known competing financial interests or personal relationships that could have appeared to influence the work reported in this paper.

## Supplementary data

MMC S1 The material outlines SSA-based decomposition theory, cycle detection, ACF/PACF plots, and trend analyses. MMC S1
