# Supplementary material for: A temporal network analysis of drug co-prescription during antidepressants and anxiolytics dispensing in the Netherlands from 2018 to 2022
Source: Glob Epidemiol. 2026 Jan 14;11:100248. doi: 10.1016/j.gloepi.2026.100248 (PMC12859469; doi:10.1016/j.gloepi.2026.100248)
Supplement: MMC S1 — The material outlines SSA-based decomposition theory, cycle detection, ACF/PACF plots, and trend analyses. [file mmc1.pdf]

Supplementary: A temporal network analysis of drug co-prescription around antidepressants and anxiolytics uses in the Netherlands from 2018 to 2022

S1 Methods

S1.1 Exploring seasonality in the dataset

Exploration on seasonality was done on detrended daily and weekly data by generating seasonal plots and calculating the autocorrelation (ACF) and partial autocorrelation function (PACF). Seasonal plots were generated for weekly and yearly pattern by using daily and weekly data, respectively. To generate the seasonal plots, the data was first deconstructed based on its period. For daily data, the weekly period was used; while for weekly data, the yearly period was used. The weekly period was obtained by creating an ordered value formatted as year - week, e.g. `2018 - W01`, whereas the yearly period was an order from 2018 to 2022. We then grouped the series by its deconstructed period and visually examine seasonality as overlapping pattern in most periods. To substantiate the findings, ACF and PACF plots were used to check on statistical significance of a given pattern.

S1.2 Decomposition with singular spectrum analysis

Classical and Seasonal-Trend decomposition technique may not sufficiently capture complex periodic patterns in a time-series. Singular spectrum analysis (SSA) is a powerful non-parametric technique by leveraging Hankel matrix as a higher-dimensional embedding of the time-series. The resulting Hankel matrix X has the size of $L \times K$, where L represents the lag term. The lag term is the length of data point in a time-series being taken to construct the higher-dimensional embedding. For a weekly data with a hypothesized yearly seasonality, the L is set as 52, representing the number of week in a year. In theory, the number of L should be between the range of $2 \leq L \leq \frac{N}{2}$, where N is the total length of the time-series (Golyandina and Zhigljavsky 2020).

$$\tau = \overset{\text{Time-series data}}{\begin{bmatrix} x_1 & x_2 & \cdots & x_{N-1} & x_N \end{bmatrix}} \quad \rightarrow \quad X = \overset{\text{Time-series embedding}}{\begin{bmatrix} x_1 & x_2 & \dots & x_{N-L+1} \\ x_2 & x_3 & \dots & x_{N-L+2} \\ \vdots & \vdots & \ddots & \vdots \\ x_L & x_{L+1} & \dots & x_N \end{bmatrix}} \tag{1}$$

The embedded time-series X is then decomposed using a singular value decomposition (SVD). With a matrix $S = X \cdot X^T$, we can extract an eigenvalue $\lambda \ni \{\lambda_1 \geq \cdots \geq \lambda_L\}$, where each λ_i is a non-negative integer. Similarly, we can define $U \ni \{U_1, \ldots, U_L\}$ as the eigenvectors of matrix S corresponding to eigenvalues λ. Afterwards, we can extract factor vectors $V \ni \{V_1, \ldots, V_L\}$ where the corresponding $V_i = X^T \frac{U_i}{\sqrt{\lambda_i}}$. Finally, the eigentriple of $\{\sqrt{\lambda_i}, U_i, V_i^T\}$ is formulated as a row-wise decomposition of matrix X (Golyandina and Zhigljavsky 2020). The resulting eigentriple is then used to reconstruct the time-series by eigentriple grouping and diagonal averaging.

In case of complex periodic patterns, sequential SSA is preferred to separate trend from seasonal components (Golyandina, Korobeynikov, and Zhigljavsky 2018). A sequential SSA model is performed by first fitting a basic SSA model to reconstruct the first eigentriple, *viz.* the eigentriple that explain most of the variance in the data. Then, the residual of the first model is extracted to fit the second SSA model. The first SSA model is defined with the lowest possible L, which in our case $L = 52$. The second SSA model is defined with the largest possible L to capture all patterns, which is conveniently defined as $L = \frac{N}{2}$. The decomposed series and its corresponding original data is then extracted from the model alongside all of the oscillating functions which contributes to complex seasonality. The oscillatory components extracted from SSA correspond to harmonics, namely a distinct periodic signals with different frequencies. These harmonics form complex seasonality and captured in pairs of eigentriples. The original data, trend, residuals, and oscillating functions are plotted for further inspection. The original data and trend is further evaluated using Mann-Kendall trend test.

S2 Results

S2.1 Cyclical patterns on de-trended data

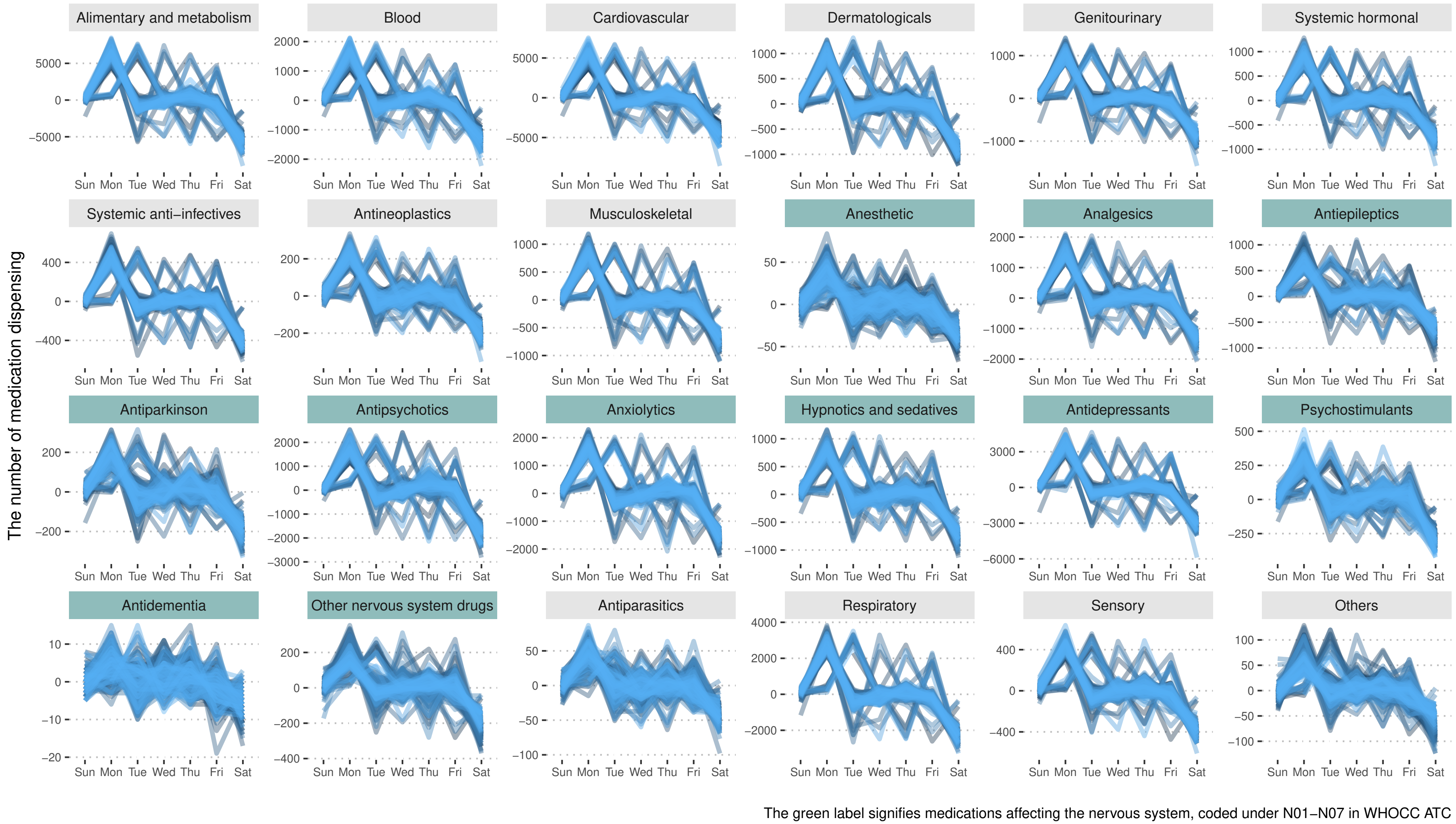

Figure 1: Daily cyclical pattern captured on a de-trended data

S2.2 Daily ACF and PACF plots

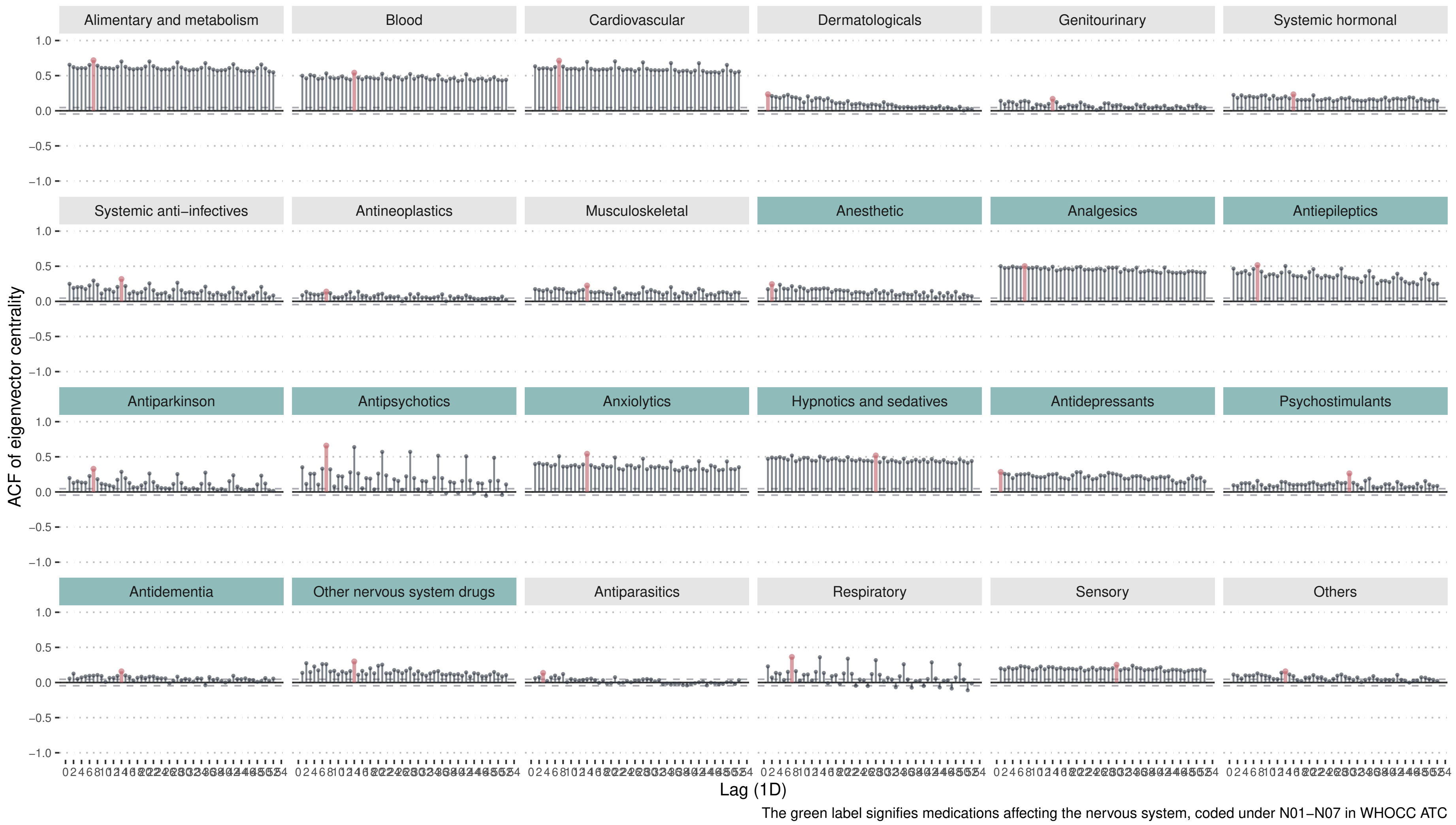

Figure 2: Daily cyclical pattern captured on a de-trended data

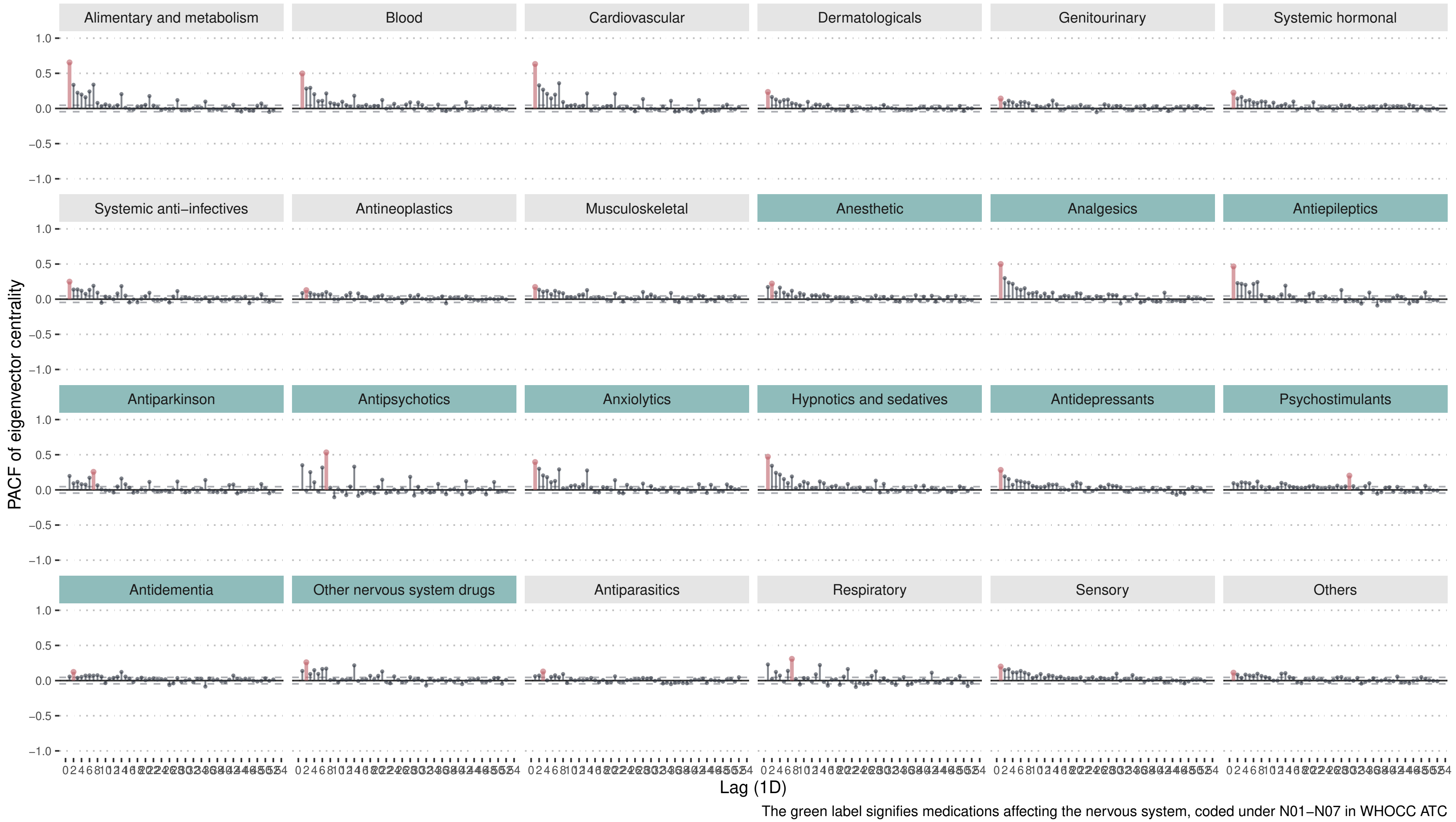

Figure 3: Daily cyclical pattern captured on a de-trended data

S2.3 Weekly ACF and PACF plots

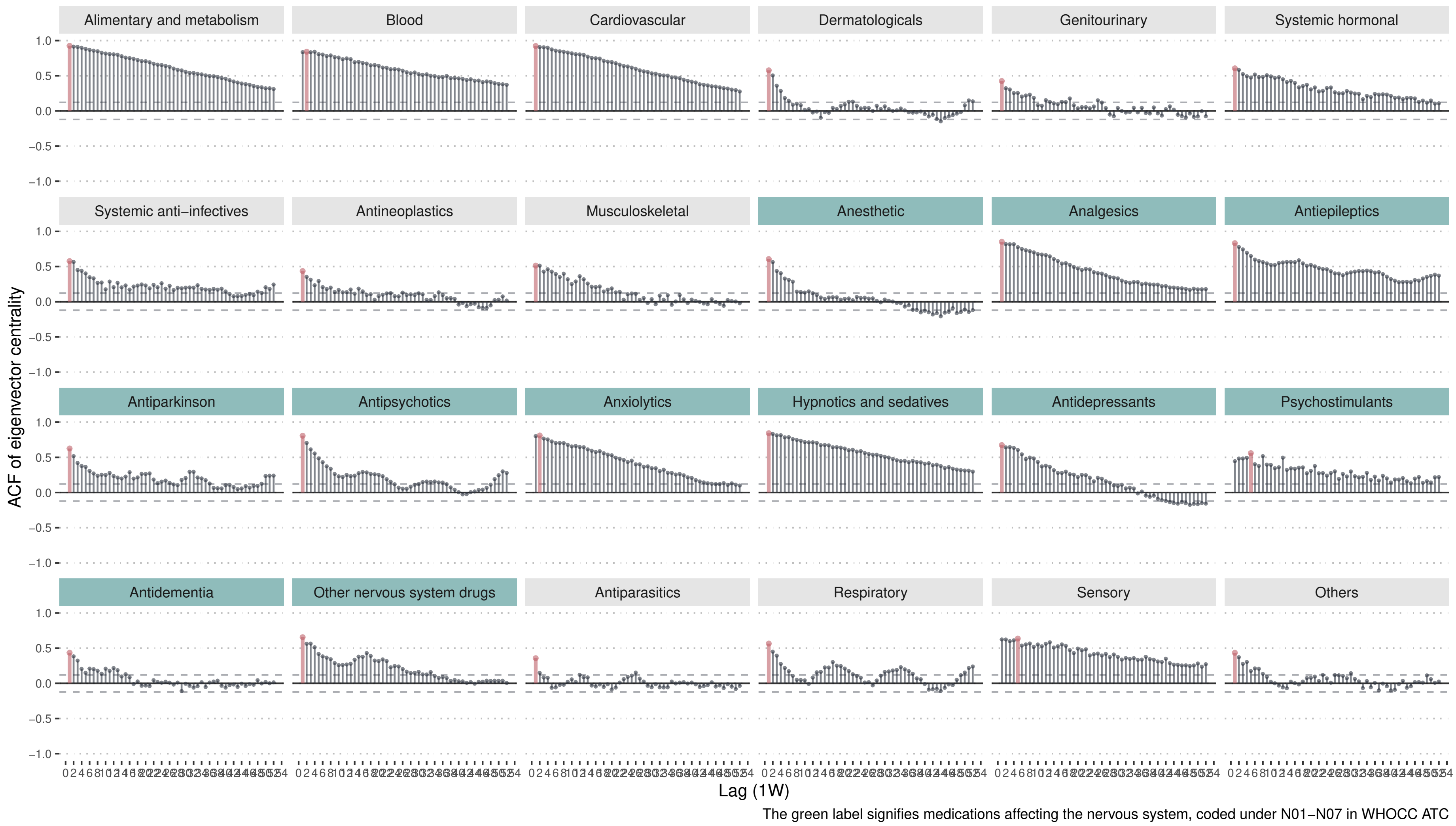

Figure 4: Daily cyclical pattern captured on a de-trended data

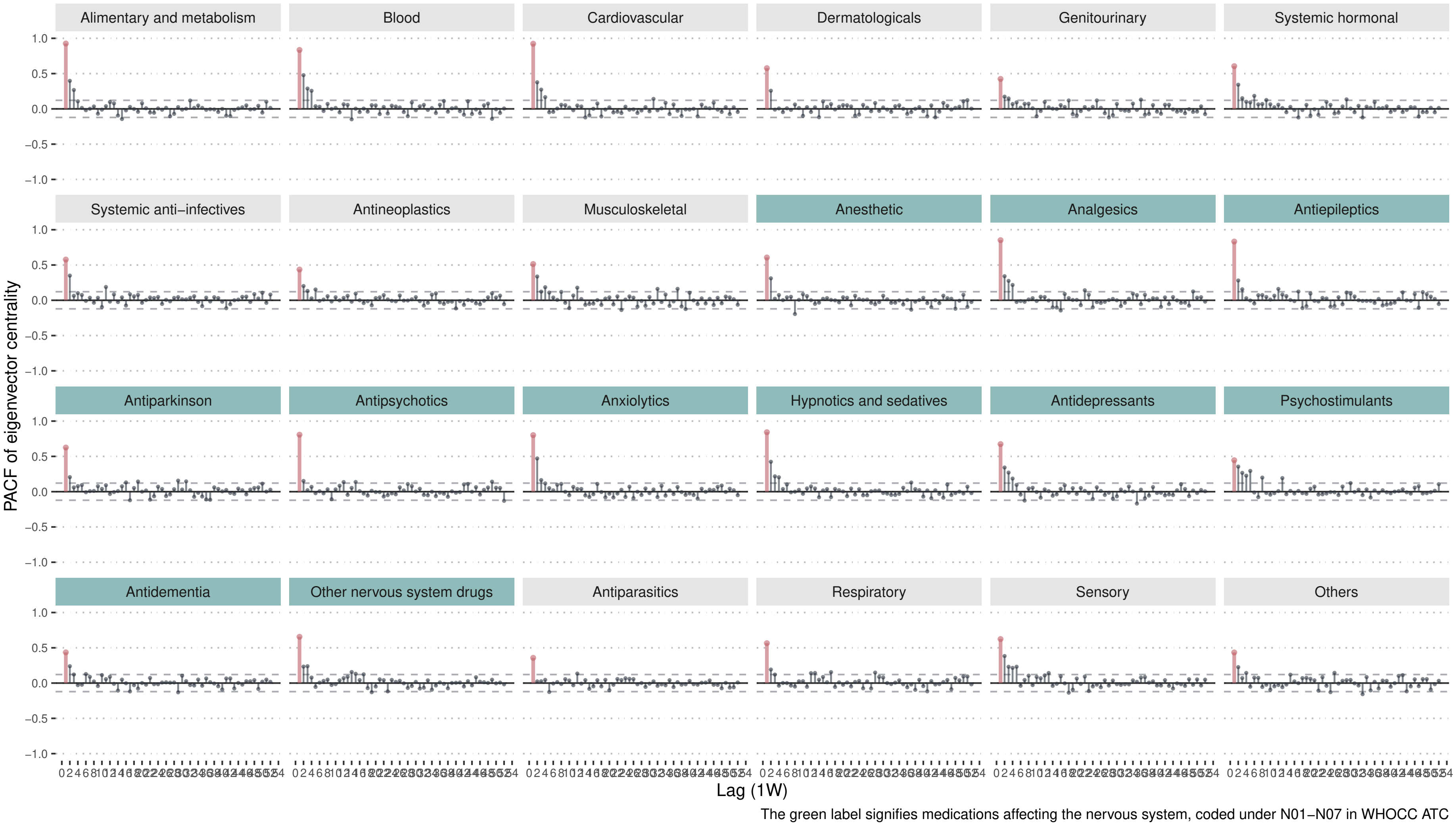

Figure 5: Daily cyclical pattern captured on a de-trended data

S2.4 Time-series decomposition of medication dispensing records

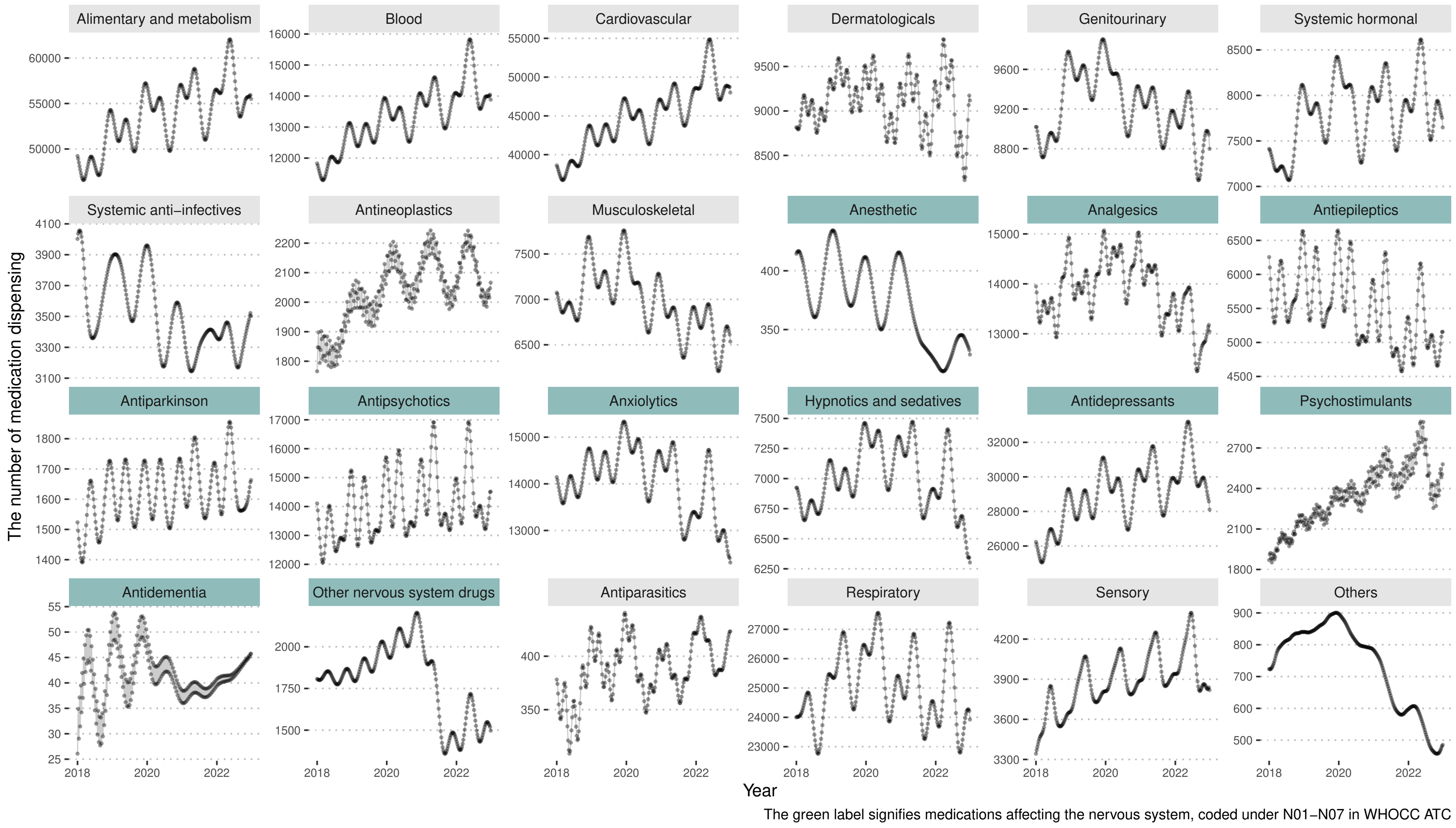

Figure 6: Reconstructed time series data using the SSA-based approach

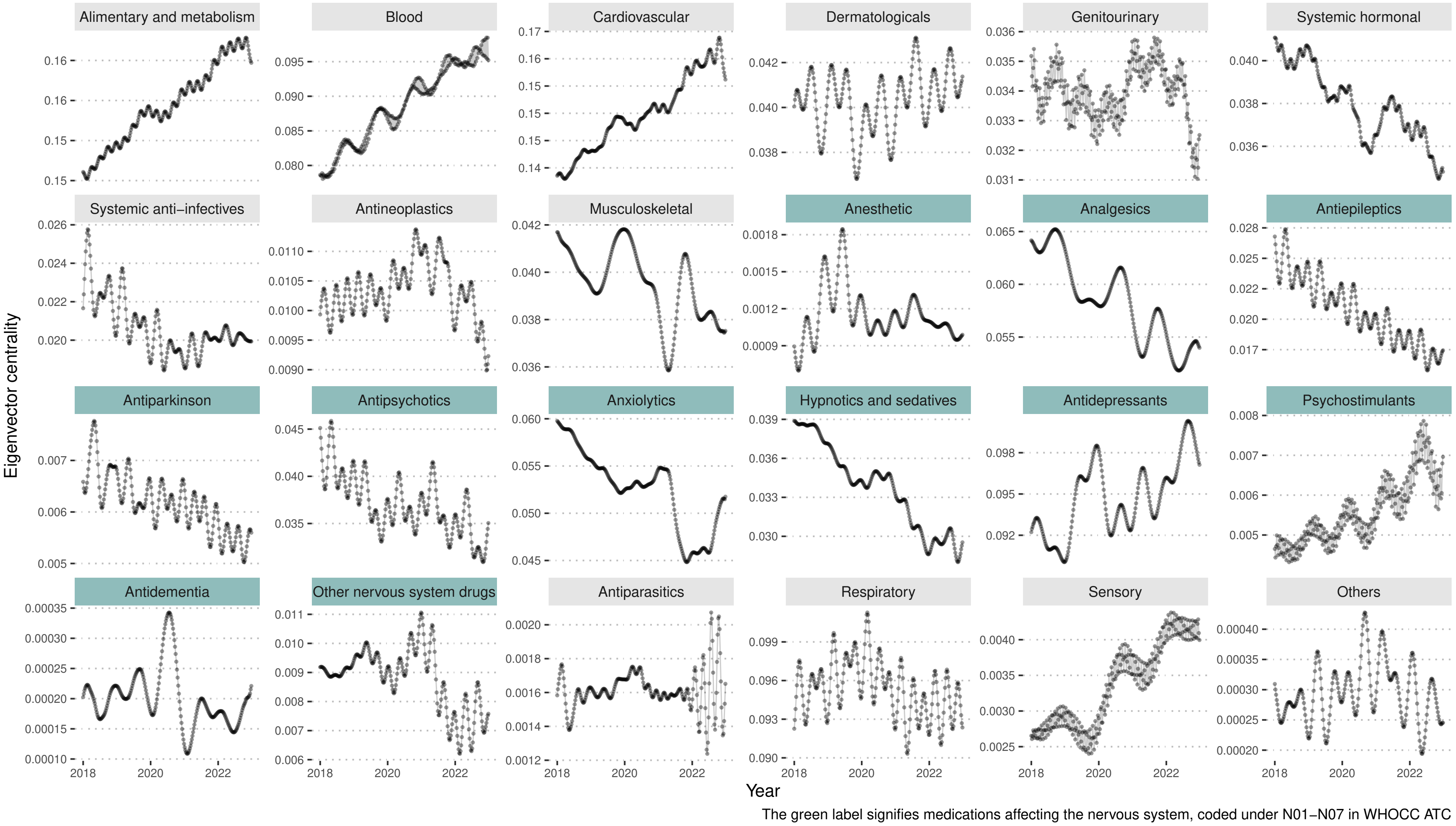

Figure 7: Reconstructed time series data using the SSA-based approach

Table 1: Trend of medication uses alongside its measure of relative importance

	Eigenvector Centrality		Prescription Dispensing	
ATC Group	Original	Trend	Original	Trend
Alimentary and metabolism	18.63 (p <0.001)	24.05 (p <0.001)	11.11 (p <0.001)	23.8 (p <0.001)
Blood	17.02 (p <0.001)	24.06 (p <0.001)	13.24 (p <0.001)	24.02 (p <0.001)
Cardiovascular	18.11 (p <0.001)	24.06 (p <0.001)	14.39 (p <0.001)	23.19 (p <0.001)
Dermatologicals	3.47 (p 0.001)	9.83 (p <0.001)	-0.1 (p 0.919)	-2.54 (p 0.011)
Genitourinary	-0.37 (p 0.714)	-1.27 (p 0.204)	-3 (p 0.003)	-11.57 (p <0.001)
Systemic hormonal	-11.44 (p <0.001)	-20.17 (p <0.001)	5.36 (p <0.001)	13.01 (p <0.001)
Systemic anti-infectives	-7.25 (p <0.001)	-12.64 (p <0.001)	-7.3 (p <0.001)	-12.25 (p <0.001)
Antineoplastics	1.22 (p 0.224)	3.58 (p <0.001)	8.86 (p <0.001)	18.52 (p <0.001)
Musculoskeletal	-6.92 (p <0.001)	-13.76 (p <0.001)	-6.6 (p <0.001)	-14.98 (p <0.001)
Anesthetic	-1.27 (p 0.204)	-10.01 (p <0.001)	-8.17 (p <0.001)	-19.57 (p <0.001)
Analgesics	-14.12 (p <0.001)	-22.7 (p <0.001)	-2.97 (p 0.003)	-4.62 (p <0.001)
Antiepileptics	-15.08 (p <0.001)	-24.07 (p <0.001)	-7.31 (p <0.001)	-17.78 (p <0.001)
Antiparkinson	-9.09 (p <0.001)	-23.07 (p <0.001)	4.27 (p <0.001)	18.66 (p <0.001)
Antipsychotics	-9.36 (p <0.001)	-17.68 (p <0.001)	6.51 (p <0.001)	18.26 (p <0.001)
Anxiolytics	-13.49 (p <0.001)	-20.76 (p <0.001)	-4.77 (p <0.001)	-9.75 (p <0.001)
Hypnotics and sedatives	-16.68 (p <0.001)	-24.06 (p <0.001)	0.13 (p 0.898)	0.58 (p 0.563)
Antidepressants	8.06 (p <0.001)	15.65 (p <0.001)	9.43 (p <0.001)	21.52 (p <0.001)
Psychostimulants	11.2 (p <0.001)	23.93 (p <0.001)	13.75 (p <0.001)	23.85 (p <0.001)
Antidementia	-0.71 (p 0.477)	-4.24 (p <0.001)	2.57 (p 0.01)	1.43 (p 0.153)
Other nervous system drugs	-7.97 (p <0.001)	-6.6 (p <0.001)	-5.85 (p <0.001)	-6.96 (p <0.001)
Antiparasitics	0.38 (p 0.706)	7.99 (p <0.001)	4.81 (p <0.001)	13.23 (p <0.001)
Respiratory	-2.57 (p 0.01)	-5.72 (p <0.001)	-0.28 (p 0.778)	-3.53 (p <0.001)
Sensory	12.14 (p <0.001)	21.08 (p <0.001)	8.15 (p <0.001)	23.46 (p <0.001)
Others	-0.87 (p 0.385)	3.77 (p <0.001)	-12.18 (p <0.001)	-14.55 (p <0.001)

References

Golyandina, Nina, Anton Korobeynikov, and Anatoly Zhigljavsky. 2018. *Singular Spectrum Analysis with r. Use R!* Springer Berlin Heidelberg. https://doi.org/10.1007/978-3-662-57380-8.

Golyandina, Nina, and Anatoly Zhigljavsky. 2020. *Singular Spectrum Analysis for Time Series. SpringerBriefs in Statistics*. Springer Berlin Heidelberg. https://doi.org/10.1007/978-3-662-62436-4.
